# Supplementary material for: Willingness of the Jordanian Population to Receive a COVID-19 Booster Dose: A Cross-Sectional Study
Source: Vaccines (Basel). 2022 Mar 9;10(3):410. doi: 10.3390/vaccines10030410 (PMC8950968; doi:10.3390/vaccines10030410)
Supplement: Supplementary file 1 [file vaccines-10-00410-s001.zip › vaccines-1612046-supplementary.pdf]

Supplementary Files

Table S1. The English translation of the questionnaire used in the study

**1-Have you received the complete vaccination course (two doses)?**
• **Yes**<br>• **No (Automatic submission)**
**2-Are you currently living in Jordan?**
• **Yes**<br>• **No (Automatic submission)**
**3-Are 18 or above ?**
• **Yes**<br>• **No (Automatic submission)**
PART ONE (DEMOGRAPHICS)
**4-What is your age?**
• 18-29 years<br>• 30-39 years<br>• 40-49 years<br>• 50-59 years<br>• 60 years or more
**5-Sex**
• Male (go to question 7)<br>• Female
**6- Are you pregnant?**
• Yes<br>• No
**7-Do you have children?**
• No<br>• Yes
**8-Do you Smoke?**
• Yes<br>• No
**9-Marital status**
• Married<br>• Other
**10-Please choose the most similar shape to your body**

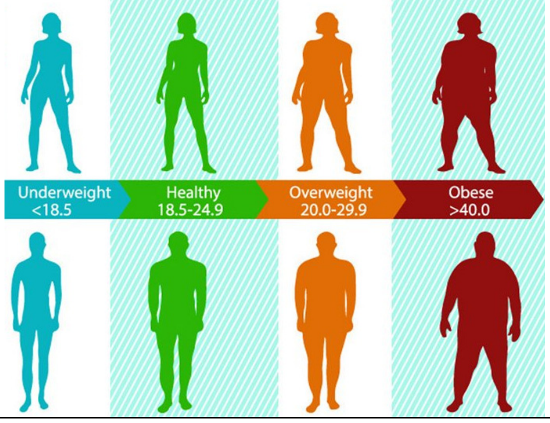

- Blue
- Green
- Orange
- Brown

11-Your weight in Kg is ________________________________

12-Your Height in cm is ________________________________

13-Education level

- High school or less
- University student
- Diploma
- Bachelor's degree
- Postgraduate

14-Area of Residency

- North region
- South region
- Zarqa
- Middle region
- Amman
- Other

15-Do you suffer from a chronic disease?

- Yes
- No (go to question 8)

16-Do you suffer from any of the following conditions: Type 2 diabetes mellitus/Chronic Obstructive Pulmonary Disease (COPD)/Cancer/Kidney Failure/Heart diseases/Organ transplantation/Sickle Cell Anemia

- Yes
- No

17-Do you suffer from any of the flowing conditions: Type 1 diabetes mellitus/Hypertension/Bone marrow transplant/ Cerebrovascular diseases or stroke/ Cystic Fibrosis/Asthma/Taking steroids or immunosuppressant drugs/Hepatic diseases/ Thalassemia/Lung fibrosis.

- Yes
- No

18-Household average monthly income

- Less than 500 JD
- 500-1000 JD
- More than 1000 JD

PART TWO (PERCEPTIONS OF COVID-19)

19-In your opinion, how serious is COVID-19?

- 1(not serious)
- 2
- 3
- 4
- 5 (extremely serious)

20-Have you ever been infected woth COVID-19?

- Yes
- Maybe
- No

21-Do you know anyone who has died due to COVID-19?

- **Yes**
- **No**

PART THREE (Knowledge of COVID-19, and practices to protect against COVID-19 infection)

**22- What are the symptoms of COVID19?**	Yes	Maybe	No
Fever			
Chills			
Diarrhea			
Cough			
Otitis media			
Loss of smell and taste			
No symptoms			

**23- How is COVID19 transmitted?**	Yes	Maybe	No
Drinking unclean water			
Eating unclean food			
Inhalation of respiratory droplets from an infected person			
Eating or touching wild animals			
Medical herb consumption			
**24- What procedure do you think that may prevent COVID19 infection?**	Yes	Maybe	No
Wearing face masks			
Washing hands with regular soap			
Using detergents			
Social distancing			
Avoiding touching face/mouth/nose/eyes			
**25-Management of COVID-19**	Yes	Maybe	No
Is there currently a drug in pharmacies or hospitals that treats COVID-19 completely?			

**26-How often do you adhere to the following protective practices against COVID-19**	Always	Most of the times	Some times	Rarely	Never
Wearing face masks					
Washing hands with regular soap					
Social distancing					
Avoiding touching face/mouth/nose/eyes					
Using detergent					

**PART FOUR (Previous COVID-19 vaccination)**
**27-In your opinion, how severe were the symptoms you experienced due to COVID-19 vaccine?**
• Mild<br>• Moderate<br>• Severe<br>• No symptoms
**28-Did you take the COVID-19 vaccine out of conviction, or were you forced by laws imposed by the state?**

- I took it out of conviction
- I took it out of conviction and because of the laws
- I took it because of the imposed laws, not because I believe in it

29-Which COVID-19 vaccine type did you receive?

- Pfizer
- AstraZeneca
- Sinopharm

30-Which side-effects did you suffer from due to COVID-19 vaccine? (You can choose more than one)

- Headache
- Hyperthermia
- Pain at site of injection
- Muscle pain
- Weakness
- Spasm
- Nausea
- Rash
- Chills
- Water retention
- No symptoms

PART FIVE (Knowledge about the booster dose)

**31-Is it necessary for the booster dose of the vaccine to be of the same type of vaccine used in the initial doses?**	**Yes**	**Maybe**	**No**

32-What is the protective time period for that the vaccine provides against COVID-19?

- Several Months
- A year
- Several Years
- Forever

33-Which people need the booster dose?

- The elderly
- People with immunosuppresent diseases
- People with cardiovascular disease
- People with renal disease
- People with pulmonary diseases
- Everybody should receive the booster dose unless contraindicated (Allergic reactions)

PART SIX (Intention to receive COVID-19 vaccine booster dose)

34-Are you willing to receive the booster dose for COVID-19 vaccine?

• Yes (Automatic submission)<br>• No<br>• May		
**35- Which of the following reasons make you hesitant towards receiving the COVID-19 booster dose?**	**Yes**	**No**
• The booster dose will not provide me with any further protection against COVID-19.<br>• The booster dose will have severe side effects.<br>• I can't tolerate another dose because the side-effects of the previous ones were severe.<br>• I was infected with COVID-19, therefore I do not need the booster dose.<br>• The symptoms I experienced due to COVID-19 infection were mild; therefore, I will not receive the booster dose.<br>• Taking the booster dose now has no benefit, however I may receive it in the future.<br>• I took the last dose a short time ago, and there will be no need to take the booster dose for at least a year.<br>• The benefits of booster doses have not been scientifically proven.<br>• The booster dose is a conspiracy to boost corporate profits.<br>• A booster dose will be imposed only based on agreements reached between pharmaceutical companies and governments.		

Table S2 Knowledge towards COVID-19 and its vaccines

How is COVID19 transmitted?	Frequency (%)
Drinking unclean water	513 (56.1)
Eating unclean food	494 (54.0%)
Inhalation of respiratory droplets from an infected person	884 (96.6%)
Eating or touching wild animals	393 (43.0%)
Medical herb consumption	213 (23.3%)
**What are the symptoms of COVID19?**	
Fever	872 (95.3%)
Chills	693 (75.7%)
Diarrhea	564 (61.6%)
Cough	772 (84.4%)
Otitis media (earache)	244 (26.7%)
Loss of smell and taste	877 (95.8%)
No symptoms	704(76.9%)
**What procedures do you think that may prevent COVID19 infection?**	
Wearing face masks	849 (92.8%)
Washing hands with regular soap	810 (88.5%)
Using detergents	880 (96.2%)
Social distancing	869 (95.0%)
Avoiding touching face/mouth/nose/eyes	843 (92.1%)
**Management of COVID-19**	
Is there currently a drug in pharmacies or hospitals that treats COVID-19 completely?	661 (72.2%)
What is the protective time period that the vaccine provides against COVID-19?	638 (69.7%)
Is it necessary for the booster dose of the vaccine to be of the same type of vaccine used in the initial doses?	198 (21.6%)
Which people need the booster dose?	442 (48.3%)

Table S3. Adherence to protective practices against COVID-19

	Always-most of the time Frequency (%)	Mean (SD)
Wearing face masks	793 (86.7%)	4.46 (± 0.85)
Washing hands with regular soap	839 (91.7%)	4.65 (±0.74)
Social distancing	689 (75.3%)	4.20 (±0.97)
Avoiding touching face/mouth/nose/eyes	680 (74.4)	4.15 (±1.07)
Using detergent	806 (88%)	4.52 (±0.80)
